# Comparative Genome Analysis of the Genus *Thiothrix* Involving Three Novel Species, *Thiothrix subterranea* sp. nov. Ku-5, *Thiothrix litoralis* sp. nov. AS and “*Candidatus* Thiothrix anitrata” sp. nov. A52, Revealed the Conservation of the Pathways of Dissimilatory Sulfur Metabolism and Variations in the Genetic Inventory for Nitrogen Metabolism and Autotrophic Carbon Fixation

**DOI:** 10.3389/fmicb.2021.760289

**Published:** 2021-10-22

**Authors:** Nikolai V. Ravin, Tatyana S. Rudenko, Dmitry D. Smolyakov, Alexey V. Beletsky, Andrey L. Rakitin, Nikita D. Markov, Alexey Fomenkov, Luo Sun, Richard J. Roberts, Andrey A. Novikov, Olga V. Karnachuk, Margarita Y. Grabovich

**Affiliations:** ^1^Institute of Bioengineering, Research Center of Biotechnology of the Russian Academy of Sciences, Moscow, Russia; ^2^Department of Biochemistry and Cell Physiology, Voronezh State University, Voronezh, Russia; ^3^New England Biolabs, Ipswich, MA, United States; ^4^Department of Physical and Colloid Chemistry, Gubkin Russian State University of Oil and Gas, Moscow, Russia; ^5^Laboratory of Biochemistry and Molecular Biology, Tomsk State University, Tomsk, Russia

**Keywords:** *Thiothrix*, genome, phylogeny, comparative genomics, metagenome-assembled genome

## Abstract

Two strains of filamentous, colorless sulfur bacteria were isolated from bacterial fouling in the outflow of hydrogen sulfide-containing waters from a coal mine (*Thiothrix* sp. Ku-5) and on the seashore of the White Sea (*Thiothrix* sp. AS). Metagenome-assembled genome (MAG) A52 was obtained from a sulfidic spring in the Volgograd region, Russia. Phylogenetic analysis based on the 16S rRNA gene sequences showed that all genomes represented the genus *Thiothrix*. Based on their average nucleotide identity and digital DNA-DNA hybridization data these new isolates and the MAG represent three species within the genus *Thiothrix* with the proposed names *Thiothrix subterranea* sp. nov. Ku-5^T^, *Thiothrix litoralis* sp. nov. AS^T^, and “*Candidatus* Thiothrix anitrata” sp. nov. A52. The complete genome sequences of *Thiothrix fructosivorans* Q^T^ and *Thiothrix unzii* A1^T^ were determined. Complete genomes of seven *Thiothrix* isolates, as well as two MAGs, were used for pangenome analysis. The *Thiothrix* core genome consisted of 1,355 genes, including ones for the glycolysis, the tricarboxylic acid cycle, the aerobic respiratory chain, and the Calvin cycle of carbon fixation. Genes for dissimilatory oxidation of reduced sulfur compounds, namely the branched SOX system (*SoxAXBYZ*), direct (*soeABC*) and indirect (*aprAB*, *sat*) pathways of sulfite oxidation, sulfur oxidation complex Dsr (*dsrABEFHCEMKLJONR*), sulfide oxidation systems SQR (*sqrA*, *sqrF*), and FCSD (*fccAB*) were found in the core genome. Genomes differ in the set of genes for dissimilatory reduction of nitrogen compounds, nitrogen fixation, and the presence of various types of RuBisCO.

## Introduction

At the end of the 19th century, Sergei Winogradsky described filamentous, colorless sulfur bacteria, often present as foulings in running waters rich in hydrogen sulfide, and gave this group of microorganisms the generic name *Thiothrix*. They can accumulate elemental sulfur in the form of intracellular inclusion bodies and are characterized by the ability to form rosettes and the presence of a pronounced mucous sheath in the filaments. They were formerly known as members of the *Thiotrichaceae* family, and are now classified into three different families *Thiolineaceae*, *Thiofilaceae*, and *Thiotrichaceae* (Boden and Scott, 2018). However, only a few cultivated representatives are known in this group of microorganisms and the taxonomy of filamentous, colorless sulfur bacteria, and in particular, of the genus *Thiothrix* is poorly developed (Boden and Scott, 2018).

Members of the genus *Thiothrix* are capable of both organoheterotrophic and lithoautotrophic growth in the presence of reduced sulfur compounds as well as of mixotrophic growth under appropriate conditions. Due to the flexible sulfur, nitrogen and carbon metabolism and the ability for aerobic and anaerobic growth, these bacteria can occupy various ecological niches. As a rule, they dominate microbial populations in sulfide-rich waters, forming powerful bacterial fouling (Larkin and Shinabarger, 1983; Chernousova et al., 2010; Rossmassler et al., 2016).

The known range of habitats of the genus *Thiothrix* is now actively expanding. Metagenomic analysis of groundwater and cave lakes has shown the presence of representatives of the genus *Thiothrix* (Macalady et al., 2008; Rossmassler et al., 2016; Panova et al., 2020). Also members of this genus were found in hydrothermal marine ecosystems, wastewater treatment bioreactors (Mardanov et al., 2020), and as ectosymbionts of invertebrates (Konkol et al., 2010; Bauermeister et al., 2012; Li et al., 2018). However, no pure cultures were obtained from these biotopes.

There are no reliable phylogenetic markers for the delineation of species within the genus *Thiothrix* since the 16S rRNA gene sequences are highly similar. For example, for *Thiothrix lacustris* BL^T^, *Thiothrix fructosivorans* Q^T^, and *Thiothrix caldifontis* G1^T^ these sequences are 98.6–98.9% identical. Therefore, the use of genome-based data like average nucleotide identity (ANI), average amino acid identity (AAI), and DNA-DNA hybridization distance (dDDH) is important for distinguishing between species and would be beneficial for building taxonomy of the genus *Thiothrix*.

After the last taxonomic revision (Boden and Scott, 2018) the genus *Thiothrix* includes five species: *Thiothrix nivea* (Larkin and Shinabarger, 1983), *T. fructosivorans*, *Thiothrix unzii* (Howarth et al., 1999), *T. caldifontis*, and *T. lacustris* (Chernousova et al., 2010). In addition, two *Thiothrix* MAGs were obtained from laboratory-scale enhanced biological phosphorus removal bioreactors (Mardanov et al., 2020). These MAGs represent two new species referred to as “*Candidatus* Thiothrix moscowensis” RT and “*Candidatus* Thiothrix singaporensis” SSD2 (Mardanov et al., 2020). Therefore, obtaining new isolates and MAGs, especially from atypical habitats, could expand our knowledge of the diversity of *Thiothrix* and would allow to identify new species of this genus.

In this work we describe three new species of the genus *Thiothrix*, *Thiothrix subterranea* sp. nov. Ku-5^T^, *Thiothrix litoralis* sp. nov. AS^T^, and a new MAG representing a new candidate species “*Candidatus* Thiothrix anitrata” sp. nov. A52. We also determined complete genomic sequences for *T*. *unzii* A1^T^ (ATCC 49747^T^) and *T*. *fructosivorans* Q^T^ (ATCC 49748^T^), that were isolated earlier (Howarth et al., 1999), but have not been sequenced until now. In addition, we performed comparative genome analysis of nine *Thiothrix* species to obtain an in-depth comparison of the metabolic potential of representatives of this genus.

## Materials and Methods

### Culture Media

Bacteria were cultivated in a medium containing (gL^–1^): (NH_4_)_2_SO_4_ – 0.5, NaNO_3_ – 0.3, CaCl_2_ – 0.03, KH_2_PO_4_ – 0.01, K_2_HPO_4_ – 0.022, Na_2_HPO_4_.7H_2_O – 0.035, MgSO_4_.7H_2_O – 0.05. Prior to inoculation, the following components were added to one liter of the medium as sterile solutions: trace elements and vitamins (Pfennig and Lippert, 1966; Kuznetsov and Dubinina, 1989), 1 ml; NaHCO_3_, 0.5 g; Na_2_S_2_O_3_.5H_2_O, 1.0 g; sodium lactate, 0.25 g and sodium acetate, 0.25 g. The pH of the medium was adjusted to 7.5. Cultures were incubated at 27°C.

### Isolation of Pure Cultures and Growth Conditions

Physiological tests were carried out using a medium of the above composition using alternative carbon sources. Three passages of culture were carried out with each of the substrates to test bacterial growth. To study the ability to fix molecular nitrogen, bacteria were cultivated in a medium of the above-described composition, excluding nitrogen sources. The ability of the strains to fix nitrogen was confirmed by the acetylene reduction assay (Stewart et al., 1968).

Isolation of pure cultures of representatives of the genus *Thiothrix* was performed from the selected bacterial fouling, where bundles of filamentous sulfur bacteria were collected, washed with a large amount of sterile water, and transferred to a Potter manual homogenizer to prepare suspensions of colorless sulfur bacteria threads, in order to obtain *Thiothrix* gonidia. A number of tenfold dilutions were prepared by the method of limiting dilution from the starting material. To obtain individual colonies, serial dilutions were plated on a solid nutrient medium of the composition described above, which additionally contained (gL^–1^): agar Difco – 7.0; Na_2_S.9H_2_O – 0.3.

Fatty acid analysis was performed as described elsewhere (Slobodkina et al., 2020). Briefly, about 10 mg of freeze-dried biomass collected at the late exponential growth phase were treated with anhydrous HCl/MeOH, extracted with hexane, and analyzed with a Trace GC Ultra DSQ II GC-MS system (Thermo Fisher Scientific, HP-5MS column, EI70 eV).

### Genome Sequencing of *T. unzii* A1^T^ and *Thiothrix* sp. AS

Genomic DNA was isolated from *Thiothrix* sp. AS and *T. unzii* A1^T^ (strain was obtained from ATCC collection ATCC 49747^T^) using a DNeasy PowerSoil DNA isolation kit (Mo Bio Laboratories, Carlsbad, CA, United States). Genomic DNA was sequenced using Illumina and Oxford Nanopore platforms. For Illumina sequencing, the shotgun genome libraries were prepared using the NEBNext Ultra II DNA library prep kit (New England Biolabs, United States). The libraries were sequenced on an Illumina MiSeq (Illumina, San Diego, CA, United States) in a paired reads mode (2 × 300 nt). Low quality sequences were trimmed using Sickle v.1.33 (*q* = 30)^1^. In addition, genomic DNA was sequenced on a MinION device (Oxford Nanopore, United Kingdom) using the ligation sequencing kit 1D and FLO-MIN110 cells. Sequencing statistics are shown in Supplementary Table 1. For each genome, nanopore reads were assembled into a single circular contig using Flye v. 2.8.2 (Kolmogorov et al., 2019). The consensus sequence of the assembled contig was corrected with two iterations of Pilon v.1.22 (Walker et al., 2014) using Illumina MiSeq reads.

### Metagenome Sequencing and Assembly of *Thiothrix* sp. A52 Genome

The total DNA was extracted from 200 μg of a microbial fouling sample using a DNeasy PowerSoil DNA isolation kit. Metagenomic DNA was sequenced using Illumina and Oxford Nanopore platforms as described above. Nanopore reads were *de novo* assembled using Flye v.2.7 (Kolmogorov et al., 2019). The obtained contigs were binned into metagenome-assembled genomes (MAGs) using MetaBAT v. 2.12.1 (Kang et al., 2015). The taxonomic assignment of the obtained MAGs was performed using the Genome Taxonomy Database Toolkit (GTDB-Tk) v.1.1.1 (Chaumeil et al., 2020) and Genome Taxonomy Database (GTDB; Parks et al., 2018). Completeness of the MAGs and their redundancy (contamination) were estimated using the CheckM v.1.05 tool (Parks et al., 2015).

One MAG, designated A52, and assigned to *Thiothrix*, was manually assembled into a single circular contig using the Flye assembly graph visualized in Bandage v. 0.8.1 tool (Wick et al., 2015). The assembly sequence was polished using Illumina reads with two iterations of Pilon v. 1.22 (Walker et al., 2014).

### Single Molecule Real Time Sequencing of *Thiothrix* sp. Ku-5 and *T*. *fructosivorans* Q^T^

Genomic DNAs from *Thiothrix* sp. Ku-5 and *T. fructosivorans* Q^T^ (strain was obtained from ATCC collection ATCC 49748^T^) were purified using a Monarch Genome Purification kit (T3010S, NEB, MA, United States) and sequenced using the Pacific Biosciences (PacBio) RSII sequencing platform. SMRTbell libraries were prepared using a modified PacBio protocol adapted for NEB reagents. Genomic DNA samples were sheared to an average size of ∼ 10–20 kb using the G-tube protocol (Covaris; Woburn, MA, United States), treated with FFPE, end repaired, and ligated with hairpin adapters. Incompletely formed SMRTbell templates were removed by digestion with a combination of exonuclease III and exonuclease VII (New England Biolabs, Ipswich, MA, United States). The qualification and quantification of the SMRTbell libraries were made on a Qubit fluorimeter (Invitrogen, Eugene, OR, United States) and a 2100 Bioanalyzer (Agilent Technologies, Santa Clara, CA, United States). Single molecule real time sequencing (SMRT) sequencing was performed using a PacBio RSII (Pacific Biosciences; Menlo Park, CA, United States) based on standard protocols for 10--20 kb SMRTbell library inserts. Sequencing reads were collected and processed using the SMRT Analysis pipeline from Pacific Biosciences^2^ (Chin et al., 2013).

In addition, genomic DNA of *T*. *fructosivorans* Q^T^ was sequenced on a GridION device (Oxford Nanopore, United Kingdom) using the ligation sequencing kit 1D and R9.4 flow cell for 12 h run. Sequencing statistics are shown in Supplementary Table 1. Nanopore reads allowed us to overcome two large, approximately 50 kb repeats and the chromosome was assembled into a single circular contig using Basecaller 4.2.3 and MinKNOW 20.10.6 software.

### Annotation and Analysis of the Genomes

Gene search and annotation were performed using the National Center for Biotechnology Information (NCBI) Prokaryotic Genome Annotation Pipeline (Tatusova et al., 2016; Haft et al., 2018). ANI was calculated using an online resource^3^ based on the OrthoANIu algorithm, using USEARCH (Lee et al., 2016). AAI between the genomes was determined using the aai.rb script from the enveomics collection (Rodriguez-R and Konstantinidis, 2016). dDDH calculation was performed using the GGDC^4^ online platform.

The core genome of analyzed strains was determined by clustering all genes selected by the BLASTclust v.2.2.26 program from the BLAST package (Korf and Gish, 2000). Clustering was carried out by the single link method. Genes whose predicted protein products had more than 70% amino acid sequence identity across more than 80% of the length were combined into one cluster. Clusters containing at least one protein from each genome formed the core genome.

### Phylogenetic Analysis

The Genome Taxonomy Database toolkit (GTDB-Tk; Chaumeil et al., 2020), release 04-RS89 was used to identify the 120 single-copy phylogenetically informative conservative marker genes used in the GTDB classification system (Parks et al., 2018) in the genomes of *T. lacustris* BL^T^, *Thiothrix* sp. AS, *T. fructosivorans* Q^T^, *Thiothrix* sp. Ku-5, *T. caldifontis* G1^T^, *Thiothrix* sp. A52, *T. unzii* A1^T^, “*Ca*. Thiothrix moscowensis RT,” “*Ca*. Thiothrix singaporensis SSD2,” *Thiothrix singaporensis* SSD2,” *Thiothrix nivea* JP2^T^, *Thiolinea disciformis* B3-1^T^, *Thiothrix eikelboomii* AP3^T^, *Thiofilum flexile* EJ2M-B^T^, and *Leucothrix mucor* DSM 2157^T^. These genes were used to construct a multiple alignment of concatenated amino acid sequences in GTDB-Tk. The multiple alignment was used to construct a maximum-likelihood phylogenetic tree in PhyML v. 3.3 (Guindon et al., 2010) with default parameters (LG amino-acid substitution model was used, equilibrium amino-acid frequencies are defined by the substitution model, gamma distribution with estimated shape parameter was used to model 4 substitution rate categories, no invariant sites). The levels of support for internal branches were assessed using the Bayesian test in PhyML.

### Nucleotide Sequence Accession Numbers

The annotated genome sequences of *Thiothrix* sp. AS, *T. unzii* A1^T^, and *Thiothrix* sp. A52 were submitted to the NCBI GenBank database and are accessible *via* BioProject PRJNA719636. The annotated genome sequences of *Thiothrix* sp. Ku-5 and *T. fructosivorans* Q^T^ were submitted to the NCBI GenBank database and are accessible *via* the BioProject PRJNA631701.

## Results

### Biotopes Harboring New Species of *Thiothrix*

**FIGURE 1:**
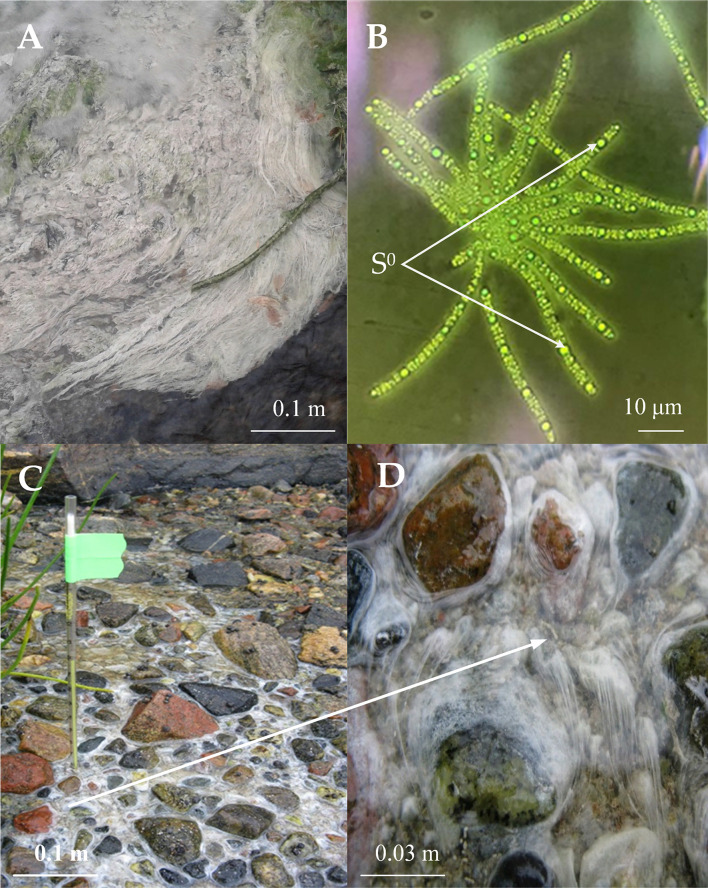
The biotope properties where *Thiothrix* samples of *Thiothrix* sp. Ku-5 **(A)** and AS **(C)** and **(D)** were collected and morphology of *Thiothrix* sp. Ku-5 cells: rosette formation; inclusion of elemental sulfur in cells **(B)**. Cell morphology was observed by using an Olympus CX41 microscope equipped with a phase-contrast device. The white arrow indicates the scaled area in **(C)** with *Thiothrix* fouling.

The samples for metagenomic analysis, as well as for the isolation of new *Thiothrix* isolates were taken from microbial fouling in the place of the outflow of sulfidic water from a drained well from the closed and flooded coal mine “Severnaya” in Kemerovo region of Russia (55°19.59′N, 84°4.59′E), a sulfidic spring in the Volgograd region of Russia (49°8.921′N, 44°6.236′E) and on the shore of the White Sea, Russia at the border of the freshwater stream flowing into the sea (66°33.17′N, 33°5.58′E) (Figures 1A,C,D). In the latter case, bacterial fouling was taken at low tide in the channel of a fresh stream; hydrogen sulfide came from the bottom sediments.

The studied biotopes were characterized by a low redox potential (from −112 to −120 mV), the oxygen concentration varied from 2 to 5 mg L^–1^, and the concentration of hydrogen sulfide was from 1 to 6 mg L^–1^. The water temperatures at all sampling sites was between 9 and 16°C (Table 1).

**TABLE 1:** Characteristics of the isolation sources.

Species or MAG	Environmental characteristics			
	T (°C)	pH	Eh (mV)	H_2_S (mg L^–^^1^)
*Thiothrix* sp. Ku-5	9.8	7.7	−112	5.4
*Thiothrix* sp. AS	9–12	7.0	−117	1–2
MAG of *Thiothrix* sp. A52	9–16	7.5	−120	4–6

### *Thiothrix* Genomes Assembly and Analysis

The *Thiothrix* sp. Ku-5 was isolated from bacterial fouling in the drainage water flowing out of the coal mine in Kemerovo, Russia. The genome sequence of the *Thiothrix* sp. Ku-5 assembled into four closed-circular contigs; one circular 4,036,668 bp long chromosome (CP053482) and three circular plasmids, – pTSUKu5_27_unt8 (CP053484), pTSUKu5_12_unt9 (CP053483), and pTSUKu5_2_unt34 (CP053485) with lengths of 26,592 bp, 12,118 bp, and 2,180 bp, respectively. Annotation of the genome of *Thiothrix* sp. Ku-5 revealed four copies of the 16S rRNA gene, 46 tRNA genes, and 3,965 potential protein-coding genes.

The *Thiothrix* sp. AS was isolated from bacterial fouling in the shore zone of the White Sea. The genome of the *Thiothrix* sp. AS was assembled into a single closed circular contig (CP072801) with a total length of 4,177,302 bp. Genome annotation revealed three copies of the 16S rRNA gene, 44 tRNA genes, and 4,045 potential protein-coding genes.

The genome of the *T. unzii* A1^T^ was assembled into eight contigs; one circular 3,648,856 bp long chromosome (CP072793.1) and seven circular plasmids: pTunz1 (CP072796.1, 34,476 bp), pTunz2 (CP072795.1, 22,458 bp), pTunz3 (CP072792.1, 21,012 bp), pTunz4 (CP072799.1, 18,228 bp), pTunz5 (CP072797.1, 14,837 bp), pTunz6 (CP072798.1, 14,317 bp), and pTunz7 (CP072794.1, 14,102 bp). The genome annotation revealed two copies of the 16S rRNA gene, 44 tRNA genes, and 3,729 potential protein-coding genes.

The original polished assembly of *T. fructosivorans* Q^T^ generated three linear contigs (1,938,437; 1,467,464 and 312,998 bp) (JAFMPM000000000.1) for the main chromosome with 51.37% GC and four closed circular plasmids pTfr153 (CP072750, 153,578 bp), pTfr21 (CP072751, 21,013 bp), pTfr12 (CP072749, 12,657 bp), pTf2 (CP072752, 2,341 bp). Therefore, *T. fructosivorans* Q^T^ was additionally sequenced on a GridION device (Oxford Nanopore, United Kingdom). Nanopore reads allowed us to overcome two large, approximately 50 kb repeats and the chromosome was finally assembled into a single circular contig (CP072748, 4,137,062 bp). Annotation of the genome of *T. fructosivorans* Q^T^ revealed two copies of the 16S rRNA gene, 44 tRNA genes, and 4,071 potential protein-coding genes.

Metagenome-assembled genome of *Thiothrix* sp. A52 was assembled from the environmental DNA isolated from bacterial fouling from a sulfidic spring in the Volgograd region. The metagenome sequencing resulted in 11 MAGs with completeness higher than 70%; three of these MAGs were assigned to the genus *Thiothrix.* One MAG was sequenced at 201-fold coverage and represented about half of the entire metagenome. Using long reads obtained by nanopore sequencing, the complete closed circular sequence of the chromosome with a length of 3,546,315 bp (CP072800) was obtained. The genome annotation revealed two copies of the 16S rRNA gene, 30 tRNA genes, and 3,613 potential protein-coding genes.

The main characteristics of the obtained genomes are shown in Table 2.

**TABLE 2:** The general properties of assembled *Thiothrix* genomes that were used for pangenome analysis.

Species	Genome assembly	Size (MB)*	Contigs	G + C content (mol %)	Proteins	16S rRNAs	tRNAs	Plasmids**
*T. lacustris* BL^T^ (DSM 21227)	GCF_000621325.1	3.72	56	51.3	3,537	2	40	U
*Thiothrix* sp. AS (VKM B-3545)	GCF_017901135.1	4.28	1	52.8	4,045	3	44	0
*Thiothrix* sp. Ku-5 (VKM B-3544)	GCF_016772315.1	4.08	4	51.1	3,885	4	46	3
*T. caldifontis* G1^T^ (DSM 21228)	GCF_900107695.1	3.94	72	50.6	3,752	1	42	U
*T. unzii* A1^T^ (ATCC 49747)	GCA_017901175.1	3.72	8	50.8	3,626	2	45	7
*T. nivea* JP2^T^ (DSM 5205)	GCF_000260135.1	4.69	15	54.9	4,327	2	44	U
*T. fructosivorans* Q^T^ (ATCC 49748)	GCA_017349355.1	4.35	6	51.3	3,616	2	44	5
“*Candidatus* Thiothrix moscowensis” RT	GCA_016292235.1	3.69	78	53.6	3,483	1	38	U
“*Candidatus* Thiothrix singaporensis” SSD2	GCA_013693955.1	4.54	1	55.6	4,097	2	43	U
MAG of *Thiothrix* sp. A52	GCF_017901155.1	3.55	1	50.1	3,387	2	45	U

* All contigs; ** U, unknown in cases of MAGs and assemblies consisting of multiple contigs.

### Phylogenetic Analysis of the Genus *Thiothrix*

**FIGURE 2:**
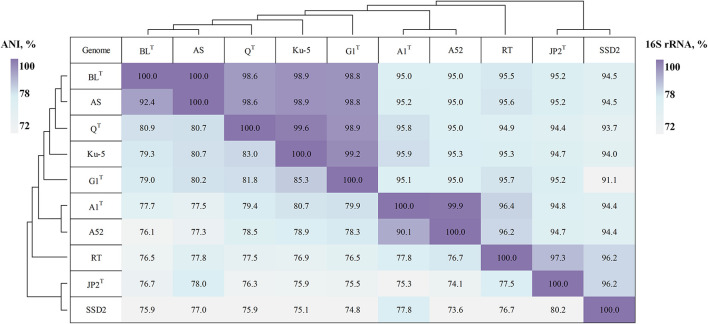
The heat map of 16S rRNA and ANI pairwise values for assembled *Thiothrix* genomes. *T. lacustris* BL^T^, (GCF_000621325.1); *Thiothrix* sp. AS (GCF_017901135.1); *T. fructosivorans* Q^T^ (GCA_017349355.1); *Thiothrix* sp. Ku-5 (GCF_016772315.1); *T. caldifontis* G1^T^ (GCF_900107695.1); *T. unzii* A1^T^ (GCA_017901175.1); MAG of *Thiothrix* sp. A52 (GCF_017901155.1); “*Ca.* Thiothrix moscowensis” RT (GCA_016292235.1); *T. nivea* JP2^T^ (GCF_000260135.1); “*Ca.* Thiothrix singaporensis” SSD2 (GCA_013693955.1).

The sequence identities of the 16S rRNA genes among the members of the genus *Thiothrix* are in the range from 93.3 to 98.9% (Figure 2). For example, the 16S rRNA genes of *Thiothrix* sp. AS and *T. lacustris* BL^T^ are completely identical (Figure 2). Strain AS previously was used as an example of the ability of representatives of the genus *Thiothrix* to grow anaerobically on nitrates (Trubitsyn et al., 2014). However, the ANI and dDDH values clearly show that *Thiothrix* sp. AS and *T. lacustris* BL^T^ are different species. According to ANI and dDDH data *Thiothrix* sp. AS is sufficiently distant from other *Thiothrix* species (<92.3% and <56.2%, respectively) to be classified as a new species (Figure 2 and Supplementary Figure 1). The ANI values between MAG *Thiothrix* sp. A52, *Thiothrix* sp. Ku-5, and other *Thiothrix* genomes are less than 90.1%, which is below the species boundary cutoff of 95% (Konstantinidis and Tiedje, 2005; Figure 2). These data are also consistent with the results of dDDH (<46.9%) (Figure 2).

**FIGURE 3:**
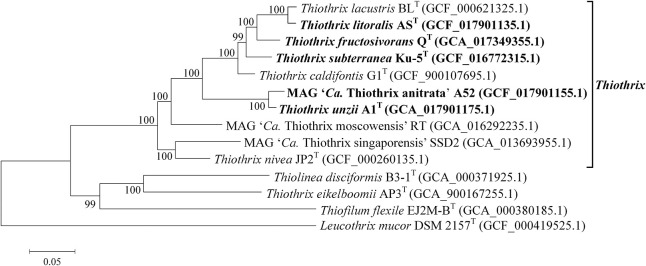
Phylogenetic tree of *Thiothrix* species. Position of *Thiothrix* genomes in the maximum-likelihood are determined by concatenated protein phylogeny. GenBank assembly accession numbers are shown after the genome names. The levels of support for internal branches assessed using the Bayesian test in PhyML are indicated at the nodes. The genome of *Leucothrix mucor* DSM 2157^T^ was used to root the tree. Note that *Thiothrix eikelboomii* actually belongs to the genus *Thiolinea* (Boden and Scott, 2018) but was not yet formally reclassified. The species whose genomes are described in this work are highlighted in bold.

The phylogenetic position of the new *Thiothrix* bacteria was also analyzed by building a phylogenetic tree based on concatenated sequences of 120 conservative marker genes. All new genomes appeared to form a distinct lineage within the genus *Thiothrix*, along with previously described species (Figure 3).

Overall, genome-based phylogenetic reconstructions and pairwise ANI and dDDH values clearly confirmed that *Thiothrix* sp. AS, *Thiothrix* sp. Ku-5, and MAG of *Thiothrix* sp. A52 can be classified as new species within the genus *Thiothrix*, for which we propose names *Thiothrix litoralis* sp. nov. AS^T^, *Thiothrix subterranea* sp. nov. Ku-5^T^, and “*Candidatus* Thiothrix anitrata” sp. nov. A52.

### Phenotypic and Physiological Properties of the New Species of *Thiothrix*

*Thiothrix* sp. Ku-5 and *Thiothrix* sp. AS are Gram negative rods forming immobile filaments (trichomes) surrounded by a mucous sheath. They form mobile gonidia, which give white colonies, which are rosettes with a diameter of 1–3 mm (Figure 1B).

The bacteria can grow in a temperature range of 4–28°C with an optimum growth at 20–22°C. pH range 6.8–8.0, with an optimal at pH 7.4–7.5. The growth range with NaCl for *Thiothrix* sp. Ku-5 is up to 1%, however, *Thiothrix* sp. AS is able to survive up to 3% NaCl in the medium. Both are capable of lithotrophic growth in the presence of reduced sulfur compounds (H_2_S, Na_2_S_2_O_3_). When growing with thiosulfate or hydrogen sulfide, elemental sulfur accumulates in the cells (Figure 1B). They are also capable of lithoautotrophic, lithoheterotrophic, and organoheterotrophic growth. Comparative characteristics of the phenotypic and cultural properties of *Thiothrix* sp. Ku-5 and *Thiothrix* sp. AS with other phylogenetic closely related species of the genus *Thiothrix* are presented in Table 3. It should be noted that the phenotypic and cultural properties of all pure cultures of representatives of the genus *Thiothrix* are largely consistent. The composition of major fatty acids determined in this work is consistent with the data obtained earlier for other species (Chernousova et al., 2009; Supplementary Table 2 and Supplementary Figure 2).

**TABLE 3:** Comparison of phenotypic and physiological properties of *Thiothrix* species.

Characteristic		1	2	3	4	5
Cell width		1.19–1.8	0.8–2.2	0.9–2.2	0.9–2.3	1.0–1.7
Cell length		4.0–6.3	4.3–6.4	3.2–6.5	4.4–6.3	4.9–10.0
**pH for growth**						
Range		6.8–8.0	6.7–8.0	7.0–8.6	6.2–8.2	6.7–8.0
Optimum		7.4–7.5	7.4–7.5	8.0	7.0	7.6–8.0
**Temperature for growth (°C)**						
Range		4–28	4–28	7–37	5–32	5–32
Optimum		20–22	20–22	25	24	25–27
Catalase activity		−	−	−	−	−
Assimilation of nitrogen		+	+	+	−	−

Organic acid as a carbon source:	Malate	−	−	−	−	+
	Oxalate	+	−	−	+	+
	Oxaloacetate	+	−	+	+	+
	Citrate	−	−	−	+	−
	Isocitrate	−	−	−	+	+
	2–Oxoglutarate	−	−	−	+	−
	Formate	−	−	−	−	+
	Aconitate	+	−	−	+	−
	Malonate	+	+	−	+	−
	Succinate	−	+	+	+	+
	Glycolate	−	−	−	−	ND

Alcohol as a carbon source:	Propanol	−	−	−	−	ND
	Inositol	+	−	−	−	ND
	Ethanol	+	−	−	−	ND
	Butanol	+	−	−	−	ND
	Isobutanol	+	−	−	−	ND
	Glycerol	−	−	−	−	ND
	Mannitol	+	−	−	−	ND
	Sorbitol	+	−	−	−	ND

Carbohydrates as a carbon source:	L–Arabinose	+	−	−	−	+
	Raffinose	+	−	−	−	+
	Sucrose	−	−	−	−	+
	Maltose	+	+	−	−	+
	D–Fructose	+	+	−	−	+
	D–Xylose	−	−	−	−	+
	L–Rhamnose	+	−	−	−	−
	L–sorbose	+	−	−	−	ND
	Trehalose	−	+	−	−	−

Amino acid as a carbon source:	Glutamate	−	+	−	+	−
	Aspartate	−	+	+	+	−
	Cysteine	−	−	−	+	−
	Asparagine	−	+	−	+	−
	Leucine	+	+	+	−	−
	Isoleucine	+	+	+	−	−
	Histidine	+	−	−	−	−
	Phenylalanine	+	−	−	−	−
	Proline	+	−	−	−	−

	Polymeric organic: compound as a carbon source:					
	Starch, Gelatin	−	−	−	−	ND
	Peptone	+	+	−	−	−
	Yeast extract	−	+	−	−	−

	Major fatty acids:					
	C_16:1_ɷ7, C_16:0_, C_18:1_ɷ7	+	+	+	+	+

*Strains: 1, *T. subterranea* Ku-5^*T*^; 2, *T. litoralis* AS^*T*^; 3, *T. caldifontis* G1^*T*^; 4, *T. lacustris* BL^*T*^; 5, *T. fructosivorans* Q^*T*^. Data were taken from this study and from Howarth et al. (1999) (*T. fructosivorans*), Chernousova et al. (2009) (*T. caldifontis*, *T. lacustris*). All strains are capable to use fumarate, lactate, acetate, pyruvate as a carbon source and are not capable to use formate, glyoxylate; methanol, propanol, glycerol; lactose, D–glucose, D–galactose, D–mannose; serine, lysine, tryptophan, methionine, tyrosine, ornithine, glutamine, alanine. +, Positive; −, negative; ND, no data available.*

According to polyphase analysis, the new isolates of *Thiothrix* sp. Ku-5 and *Thiothrix* sp. AS were assigned as new species of the genus *Thiothrix*, *T. subterranea* sp. nov. Ku-5^T^, and *T. litoralis* sp. nov. AS^T^, respectively.

#### Description of *Thiothrix subterranea* sp. nov.

*Thiothrix subterranea* (sub.ter.ra′ne.a. L. fem. adj. *subterranea* underground, below the earth surface, from where the strain was isolated). The cell size is 1.19–1.8 × 4.0–6.3 μm. Growth is observed in the temperature range 4–28°C with an optimum of growth at 20–22°C, pH 6.8–8.0 with an optimum at pH 7.4–7.5. It can tolerate NaCl up to 1%. It is catalase negative, oxidase positive, Gram negative and can sustain aerobic, microaerobic and anaerobic growth. Anaerobic growth is possible in the presence of nitrates. It is capable of all types of lithoautotrophic, lithoheterotrophic and organoheterotrophic growth. This strain uses alcohols (ethanol, butanol, isobutanol, mannitol, sorbitol, inositol), organic acids (acetate, lactate, pyruvate, aconitate, oxaloacetate, fumarate, malonate), amino acids (leucine, isoleucine, histidine, phenylalanine, proline), carbohydrates (arabinose, raffinose, maltose, fructose, rhamnose, sorbose) as carbon and energy sources, while during lithoheterotrophic growth, they are used mainly as a source of carbon. During lithotrophic growth, reduced sulfur compounds like H_2_S, Na_2_S_2_O_3_ can be used as an electron donor. It also capable of fixing molecular nitrogen. Major fatty acids are C_16:1_ɷ7, C_16:0_, C_18:1_ɷ7. The G + C content of the genome of *T. subterranea* Ku-5^T^ strain is 51.1%.

The type strain *Thiothrix subterranea* Ku-5^T^ (=VKM B-3544, =UQM 41459, =DSM 113265) was obtained from microbial fouling in the place of the outflow of drainage water from the coal mine “Severnaya,” Kemerovo, Kuzbass, Russia.

#### Description of *Thiothrix litoralis* sp. nov.

*Thiothrix litoralis* (li.to.ra′lis. L. fem. adj. *litoralis* of or belonging to the seashore). The cell size is 0.8–2.2 × 4.3–6.4 μm. Growth is observed in the temperature range 4–28°C with an optimum at 20–22°C, pH 6.7–8.0 with an optimal at pH 7.4–7.5. It can tolerate NaCl up to 3%, catalase negative, oxidase positive, Gram negative and can sustain aerobic, microaerobic and anaerobic growth. Anaerobic growth is possible in the presence of nitrates. The strain is also capable of lithoautotrophic, lithoheterotrophic and organoheterotrophic growth. It uses organic acids (acetate, lactate, pyruvate, fumarate, malonate, succinate), amino acids (glutamate, aspartate, asparagine, leucine, isoleucine), and carbohydrates (maltose, fructose, trehalose) as carbon and energy sources, while during lithoheterotrophic growth, they are used mainly as a source of carbon. During lithotrophic growth, reduced sulfur compounds like H_2_S and Na_2_S_2_O_3_ can be used as an electron donor. It is capable of fixing molecular nitrogen. Major fatty acids are C_16:1_ɷ7, C_16:0_, C_18:1_ ɷ 7. The G + C content in the genome of *T. litoralis* AS^T^ is 52.8%.

The type strain, *Thiothrix litoralis* AS^T^ (=VKM B-3545, =UQM 41458, =DSM 113264), was isolated from bacterial growths in the seashore zone of the White Sea, Murmansk region, Russia.

**“*Candidatus* Thiothrix anitrata” sp. nov.** [a.ni.tra′ta. Gr.pref. *a*, not; N.L. masc. n. *nitras* (genitive: *nitratis*), nitrate; N.L. fem.adj. *anitrata*, not reducing nitrate], MAG was obtained from the metagenome of bacterial fouling of a hydrogen sulfide brook in the Volgograd region, Russia.

### The Pangenome of the Genus *Thiothrix*

In this work, representatives of the genus *Thiothrix* were studied using a pangenomic approach. We analyzed all publicly available genomes of taxonomically described species of the genus *Thiothrix*. In addition, the newly obtained genome sequences of *T. subterranea* Ku-5^T^, *T. litoralis* AS^T^, “*Ca*. Thiothrix anitrata” A52, *T. fructosivorans* Q^T^ and *T. unzii* A1^T^ were included in the analysis (Table 2). All genomes were of high quality except for MAG of “*Ca*. Thiothrix singaporensis” SSD2 which contained multiple frameshifts and therefore was excluded from the analysis.

**FIGURE 4:**
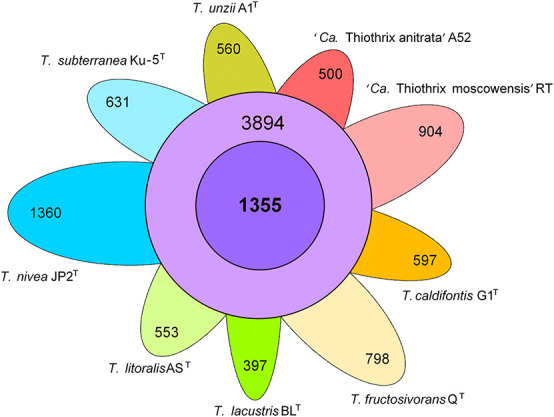
Pangenome of the genus *Thiothrix*. Venn diagram demonstrating the distribution of common (purple), additional (violet), and unique (different colors) genes among analyzed *Thiothrix* species.

The pangenome analysis of nine species indicated that the genus *Thiothrix* comprises 11,549 gene clusters. The core genome of the genus *Thiothrix* contains 1,355 genes and the auxiliary genome comprises 3,894 gene clusters present in 2–8 genomes. The number of unique genes varies in different species from 397 to 1,360 (Figure 4). These genes were predicted to encode mostly hypothetical proteins with unknown functions, transporters, transcriptional regulators, methyltransferases, transposases, and the toxin-antitoxin systems.

The genus *Thiothrix* is characterized by the presence of a number of genes for dissimilatory sulfur metabolism. *Thiothrix* species are classical lithotrophs, capable of using reduced sulfur compounds as an energy source. The hydrogen sulfide oxidation systems, SQR (*sqrA* and *sqrF*) and FCSD (*fccAB*), are present in the core genome and were found in all species. The branched SOX system, represented by the *soxAXYZB* genes and involved in the oxidation of thiosulfate to sulfur and sulfate, was also found in all members of the genus. The genes of the reverse Dsr (rDsr) *dsrABEFHÑEMKLJONR* complex involved in the oxidation of sulfur to sulfite were also present in all species, as well as genes for direct (*soeABC*) and indirect (*aprAB*, *sat*) pathways of oxidation of sulfite to sulfate. Thus, the presence of genes of several systems of dissimilatory sulfur metabolism in all known species of the genus *Thiothrix* indicates their exceptional role in the life of this group of sulfur bacteria.

The core genome contains the *hyaA* and *hyaB* genes encoding the large and small catalytic subunits of membrane-bound [NiFe] -hydrogenase of type I, and the *hyaC* gene, which encodes the cytochrome *b* subunit, which attaches hydrogenase to the cytoplasmic membrane. In addition, the core genome contains *hypABCDEF* genes, which are involved in the maturation of [NiFe] – hydrogenases.

The core genome includes all genes for the glycolysis, the Krebs cycle and the glyoxylate cycle. All members of the genus *Thiothrix* lack the classical NAD-dependent malate dehydrogenase (*mdh*), but instead contain genes encoding FAD-dependent malate: quinone oxidoreductase (*mqo*). The *glk* gene for glucokinase was found in all members of the genus, however, it was not included in the core genome due to the lower sequence similarity, which suggests horizontal transfer or an increase rate of evolution of this gene.

**FIGURE 5:**
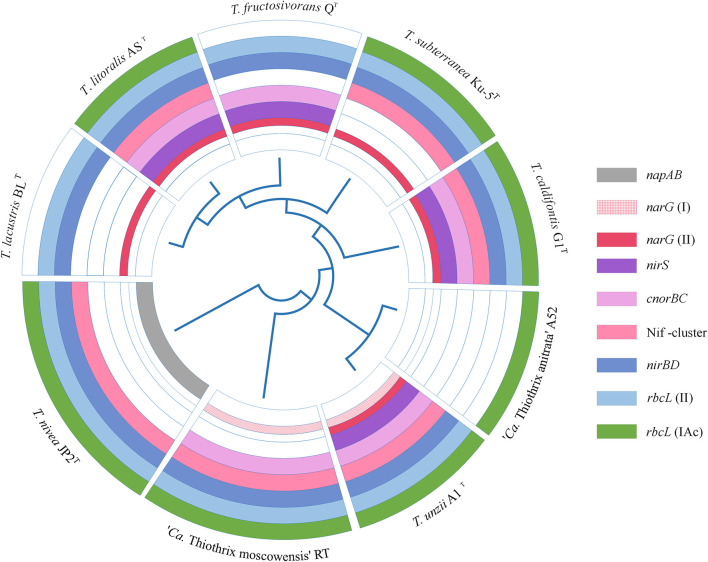
The presence of genes for nitrogen metabolism and various types of RuBisCO in the genomes of *Thiothrix* sp. Dissimilatory nitrate reduction and nitrogen assimilation genes: *napAB*, periplasmic nitrate reductase; *narG*, membrane-bound nitrate reductase; *nirS*, nitrite reductase; *cnorB*, NO-reductase; *nirBD*, ammonia forming assimilatory nitrite reductase; *nifDKH*, nitrogenase genes; *rbcL* (II) and *rbcL* (IAc), genes encoding the large subunit of RuBisCO.

All *Thiothrix* species have a complete set of genes for the Calvin-Benson-Bassham cycle of carbon fixation. Genes for the type IAq RuBisCO were in the core genome, while type IAc was found in all genomes with the exception of *T. lacustris* BL^T^ and *T. fructosivorans* Q^T^. All the species except “*Ca*. Thiothrix anitrata” A52 also have type II RuBisCO (Figure 5).

The electron transport chain (ETC) was represented in all species by complex I NuoABCDEFGHIJKLMN, complex II SdhABCD, complex III (CytB, Cyc1, Ryp1), and cytochrome *c* oxidase of complex IV (CcoNOPGQS). The presence of all ETC genes in all genomes additionally confirms the respiratory type of metabolism in the genus *Thiothrix* (Chernousova et al., 2009).

All members of the genus *Thiothrix* except for “*Ca*. Thiothrix anitrata” A52, are capable of anaerobic respiration with nitrogen compounds. However, the genes for dissimilatory nitrogen metabolism were not found in the core genome, and their set differs markedly among *Thiothrix* species (Figure 5). The *nirS* nitrite reductase gene is presented in *T*. *litoralis* AS^T^, *T*. *unzii* A1^T^, *T*. *fructosivorans* Q^T^, *T*. *caldifontis* G1^T^, but was probably lost in *T. lacustris* BL^T^, *T. nivea* JP2^T^, “*Ca*. Thiothrix moscowensis” RT, *T. subterranea* Ku-5^T^, and “*Ca*. Thiothrix anitrata” A52. Assimilatory ammonium-forming nitrite reductase *nirBD* is absent only in “*Ca*. Thiothrix anitrata” A52. The *cnorBC* nitric oxide (NO) reductase genes were found in *T*. *litoralis* AS^T^, *T*. *unzii* A1^T^, *T*. *fructosivorans* Q^T^, *T*. *caldifontis* G1^T^, and “*Ca.* Thiothrix moscowensis” RT. All members of the genus *Thiothrix* lack the *nosZ* gene encoding the dissimilatory N_2_O reductase. In the cases of *nirS, nirBD* and *cnorBC*, the corresponding genes found in different *Thiothrix* species were highly homologous; therefore, their absence in individual species probably indicates a loss in course of evolution rather than an independent acquisition as a result of horizontal gene transfer.

The distribution pattern is more complex for dissimilatory nitrate reductase genes. In the *Thiothrix* genomes two types of genes for NarGHI reductase were found, the identity of their deduced amino acid sequences is less than 60%. Genomes of *T. lacustris* BL^T^, *T*. *litoralis* AS^T^, *T*. *fructosivorans* Q^T^, *T. subterranea* Ku-5^T^, and *T*. *caldifontis* G1^T^ contain only one type of *narG*, the genome of “*Ca.* Thiothrix moscowensis” RT harbored only another type, and the genome of *T. unzii* A1^ T^ contains both variants of the *narG* gene. The *narG* genes are missing in the genomes of “*Ca*. Thiothrix anitrata” A52 and *T. nivea* JP2^T^; in the later species the periplasmic nitrate reductase *napAB* is present instead of the *narGHI* (Figure 5). Such distribution pattern indicates the events of the acquisition of additional copies of the *nar* genes as a result of horizontal transfer with the subsequent loss of one of the two copies, as well as the replacement of *nar* operon with Nap type of nitrate reductase.

The *nifASUBNXX2YB2ENQVWMHDKZTO* gene cluster for molecular nitrogen fixation is present in all species of the genus, with the exception of *T. fructosivorans* Q^T^, “*Ca*. Thiothrix anitrata” A52, and *T. lacustris* BL^T^. The amination process for all members of the *Thiothrix* genus is represented by glutamine synthetase (*glnB*), glutamate synthase (*gltBD*), and aspartate aminotransferase (*aspB*).

The genes *pstDCABS* and *phoURB*, involved in inorganic phosphorus transport into the cell, were found in the core genome. The core genome of the genus *Thiothrix* contains gene for polyphosphate kinases PPK1 that can catalyzes the synthesis of the poly-P chain. Also The core genome contains gene for poly-AMP phosphotransferase (PAP), which belongs to class II of polyphosphate kinases PPK2 and that can catalyze the synthesis of the ADF (Kimura and Kamatani, 2021). Most likely, in *Thiothrix*, PAP is responsible for ATP regeneration in the cell (Kameda et al., 2001). In addition, the *epp* gene for exopolyphosphatase, which enables the cleavage of polyphosphates, was found in all genomes. The presence of polyphosphate kinase PPK1 and exopolyphosphatase suggests that *Thiothrix* species could accumulate and degrade polyphosphates which are probably used for temporal ATP storage.

## Discussion

The genus *Thiothrix* is relatively poorly studied due to the small number of described species, the complexity of cultivation, as well as the limited number of genomic sequences and high-quality MAGs. In this work and previously (Mardanov et al., 2020) we have obtained new pure cultures of new species of the genus *Thiothrix*, including *T*. *litoralis* sp. nov. AS^T^, *T*. *subterranea* sp. nov. Ku-5^T^, and two MAGs, assigned to candidate species “*Ca*. Thiothrix moscowensis” RT, and “*Ca*. Thiothrix anitrata” A52. For the first time full genomic sequences were obtained for *T*. *unzii* A1^T^ and *T*. *fructosivorans* Q^T^, which were isolated back in 1999 (Howarth et al., 1999). Available genomes enabled us to perform comparative analysis of nine species of this genus and reveal their metabolic features.

Comparative genome analysis revealed that the key features common to all species of the genus *Thiothrix* are their ability for lithotrophic growth in the presence of reduced sulfur compounds, H_2_S, and thiosulfate, the latter being oxidized through the branched Sox pathway (*SoxAXYZB*); autotrophic carbon fixation (Calvin-Benson-Bassham cycle), and the presence of FAD-dependent malate: quinone oxidoreductase (EC 1.1.5.4) instead of the classical NAD-dependent malate dehydrogenase (EC 1.1.1.37). The ability for lithoautotrophic and lithoheterotrophic growth in the presence of reduced sulfur compounds, as well as for organoheterotrophic growth, has been previously proven experimentally (Larkin and Shinabarger, 1983; Chernousova et al., 2009).

All members of the genus *Thiothrix* have all the necessary genes encoding enzymes involved in H_2_ oxidation during lithotrophic growth. However, lithotrophic growth in the presence of H_2_ was assumed only on the basis of the genome analysis and requires experimental verification.

The main differences between the *Thiothrix* genomes included genes involved in dissimilatory reduction of nitrogen compounds and nitrogen fixation, as well as genetic determinants of various types of RuBisCO (Figure 5). Probably, oxidized forms of nitrogen that could be used in anaerobic respiration are not available in all ecosystems in which representatives of *Thiothrix* were found, which explain the patchy distribution of the corresponding genes in the genomes. Likewise, the presence of different forms of RuBisCOs in *Thiothrix* could be explained by the flexibility of the CO_2_-fixing lifestyles associated with environments where the levels of CO_2_ and O_2_ can fluctuate, and different forms of RuBisCO could be used under different environmental conditions (Badger and Bek, 2008).

## Data Availability Statement

The datasets created for this study can be found at GenBank (https://www.ncbi.nlm.nih.gov/genbank) *via* the access numbers: GCF_017901135.1, GCF_016772315.1, GCA_017901175.1, GCA_017349355.1, and GCF_017901155.1.

## Author Contributions

NR and MG: conceptualization, supervision, and funding acquisition. DS, TR, AR, NM, AF, and AN: investigation. OK: resources. NR and AB: data curation. AB, AF, LS, and RR: formal analysis. TR, DS, and NM: validation. NR, DS, TR, AF, and MG: writing – original draft preparation. NR, TR, AF, RR, and MG: writing – review and editing. All authors have read and agreed to the published version of the manuscript.

## Conflict of Interest

AF, LS, and RR are paid employees of New England Biolabs (https://www.neb.com/). There are no patents, products in development, or marketed products associated with this research to declare. This does not alter our adherence to Frontiers in Microbiology policies on sharing data and materials. The remaining authors declare that the research was conducted in the absence of any commercial or financial relationships that could be construed as a potential conflict of interest.

## Publisher’s Note

All claims expressed in this article are solely those of the authors and do not necessarily represent those of their affiliated organizations, or those of the publisher, the editors and the reviewers. Any product that may be evaluated in this article, or claim that may be made by its manufacturer, is not guaranteed or endorsed by the publisher.

## Footnotes

1
https://github.com/najoshi/sickle

2
http://www.pacbiodevnet.com/SMRT-Analysis/Software/SMRT-Pipe

3
https://www.ezbiocloud.net/tools/ani

4
https://ggdc.dsmz.de/ggdc.php#
