# Supplementary material for: Comparative Genome Analysis of the Genus Thiothrix Involving Three Novel Species, Thiothrix subterranea sp. nov. Ku-5, Thiothrix litoralis sp. nov. AS and “Candidatus Thiothrix anitrata” sp. nov. A52, Revealed the Conservation of the Pathways of Dissimilatory Sulfur Metabolism and Variations in the Genetic Inventory for Nitrogen Metabolism and Autotrophic Carbon Fixation
Source: Front Microbiol. 2021 Oct 22;12:760289. doi: 10.3389/fmicb.2021.760289 (PMC8570282; doi:10.3389/fmicb.2021.760289)

Supplementary Material

Supplementary Table S1. Sequencing statistics.

Genome or metagenome	Illumina reads	SMRT reads	Average length of Illumina reads, bp	Average length of SMRT reads, bp	Nanopore reads	Average length of Nanopore reads, bp
Thiothrix unzii A1^T (ATCC 49747) (isolate genome)	4165564	NA	268	NA	141232	7361
Thiothrix sp. AS (isolate genome)	6728708	NA	243	NA	538091	7606
Thiothrix sp. A52 (metagenome)*	4269800	NA	268	NA	702367	9648
Thiothrix sp. Ku-5 (isolate genome)	NA	264116	NA	10652	NA	NA
Thiothrix fructosivorans Q^T (ATCC 49748) (isolate genome)	NA	249026	NA	3352	156937	10910

*, number of metagenomics reads representing this MAG; NA, not applicable

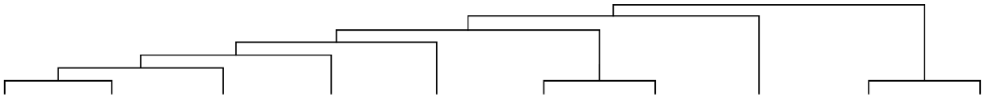

dDDH, %: 100 / 36 / 22

Genome	BL^T	AS	Q^T	Ku-5	G1^T	A1^T	A52	RT	JP2^T	SSD2
BL^T	100.0	56.2	28.8	27.7	28.0	27.8	27.7	24.9	23.9	22.4
AS	56.2	100.0	23.7	28.5	28.6	23.1	27.5	25.3	24.6	24.6
Q^T	28.8	23.7	100.0	34.7	32.7	31.8	23.7	27.8	23.7	22.2
Ku-5	27.7	28.5	34.7	100.0	38.5	36.6	35.9	27.9	23.6	22.4
G1^T	28.0	28.6	32.7	38.5	100.0	37.2	35.0	30.2	23.8	22.7
A1^T	27.8	23.1	31.8	36.6	37.2	100.0	46.9	30.5	23.4	22.9
A52	27.7	27.5	23.7	35.9	35.0	46.9	100.0	32.3	24.6	21.3
RT	24.9	25.3	27.8	27.9	30.2	30.5	32.3	100.0	25.6	24.9
JP2^T	23.9	24.6	23.7	23.6	23.8	23.4	24.6	25.6	100.0	25.6
SSD2	22.4	24.6	22.2	22.4	22.7	22.9	21.3	24.9	25.6	100.0

Supplementary Figure S1. The heat map of dDDH pairwise values for assembled *Thiothrix* genomes. *T. lacustris* BL^T, (GCF_000621325.1); *Thiothrix* sp. AS (GCF_017901135.1); *T. fructosivorans* Q^T (GCA_017349355.1); *Thiothrix* sp. Ku-5 (GCF_016772315.1); *T. caldifontis* G1^T (GCF_900107695.1); *T. unzii* A1^T (GCA_017901175.1); MAG of *Thiothrix* sp. A52 (GCF_017901155.1); '*Ca.* Thiothrix moscowensis' RT (GCA_016292235.1); *T. nivea* DSM 5205^T (GCF_000260135.1); '*Ca.* Thiothrix singaporensis' SSD2 (GCA_013693955.1).

Supplementary Table S2. Comparison of the cellular fatty acid contents of *Thiothrix* strains. Values are percentages of the total peak area.

Fatty acid	ECL*	*Thiothrix* sp. Ku-5	*Thiothrix* sp. AS	*T. unzii* A1^T	*T. lacustris* BL^T	*T. caldifontis* G1^T
8-oxo $C_{9:0}$	9.68	–	2.2	–	–	6.1
$C_{16:1}\omega7$	15.80	55.3	62.1	67.1	55.8	45.1
$C_{16:1}\omega6$	15.85	–	–	–	3.9	–
$C_{16:0}$	16.00	21.0	15.9	19.4	21.2	25.0
$C_{18:1}\omega7$	17.81	23.7	19.8	13.5	19.1	23.8

*ECL, equivalent chain length (HP-5MS column)

** The identification is tentative; mass spectrum is shown in Supplementary Figure S2 (Note that the molecular ion with the m/z=186 corresponds to the formula of $C_{10}H_{18}O_3$)

–, not detected

Supplementary Figure S2. Mass spectra of the detected methylated compounds.

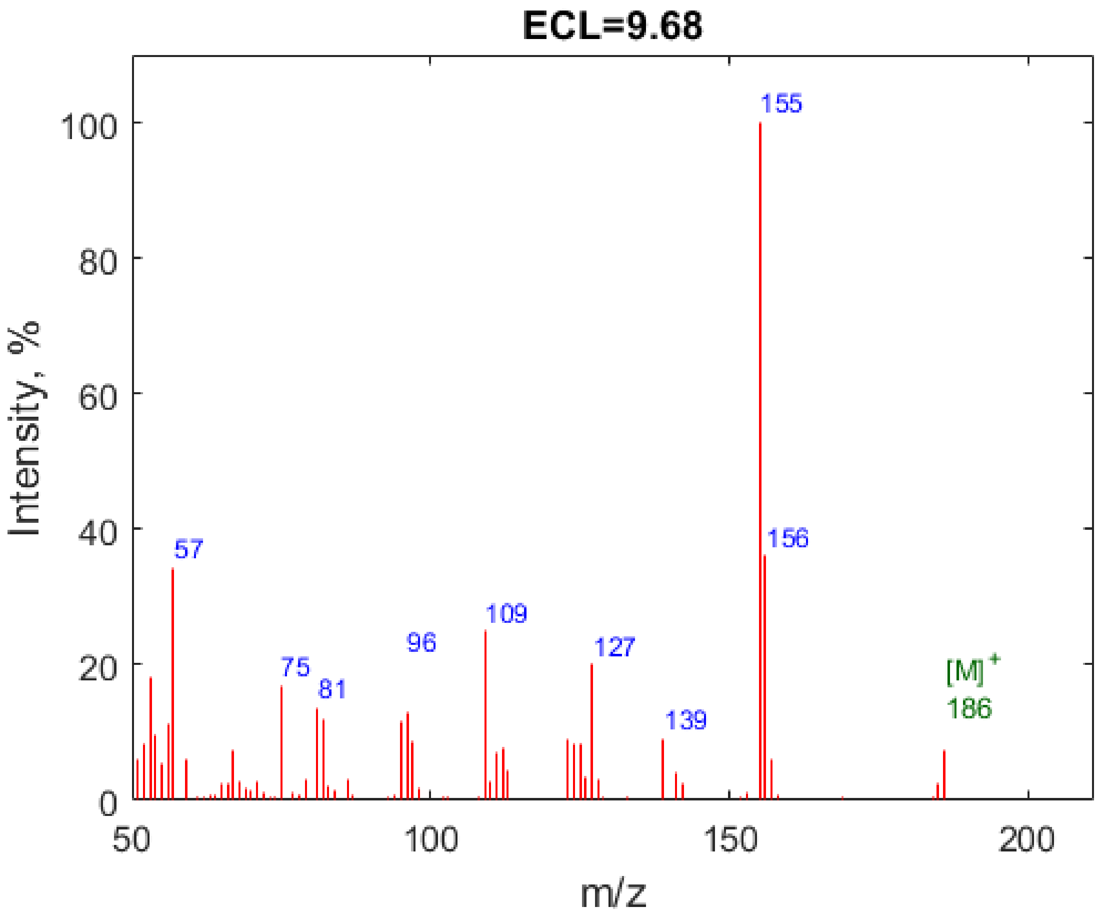

Supplement: Supplementary file 1 [file Data_Sheet_1.pdf]
